# How does the increase in foreign players affect football?

**DOI:** 10.1186/s13102-023-00643-1

**Published:** 2023-03-15

**Authors:** Ozan Sever, Erdem Ciğerci, Melih Öztop, Gökhan İpekoğlu, Süleyman Gönülateş, Zeki Akyildiz, Hadi Nobari

**Affiliations:** 1 Sports Science Faculty, Atatürk University, Erzurum, Turkey; 2 Sports Science Faculty, Kastamonu University, Kastamonu, Turkey; 3 Sports Science Faculty, Gaziantep University, Gaziantep, Turkey; 4 Sports Science Faculty, Ordu University, Ordu, Turkey; 5 Sports Science Faculty, Pamukkale University, Denizli, Turkey; 6 Sports Science Department, Gazi University, Ankara, Turkey; 7 Department of Exercise Physiology, Faculty of Educational Sciences and Psychology, University of Mohaghegh Ardabili, Ardabil, 5619911367 Iran; 8 Faculty of Sport Sciences, University of Extremadura, 10003 Cáceres, Spain

**Keywords:** Foreign players, Football performance, Football analytics, Football statistics, Turkish Super League

## Abstract

**Background:**

In the study, we tried to analyze the effects of foreign players on Turkish Super League matches. For this purpose, in this long-term study, 1836 competition data played in 6 seasons between 2014 and 2020 was obtained from www.mackolik.com each year.

**Method:**

Pearson's correlation coefficient applied between 18 different variables (goal, shoot, pass, cross, corner, offside, foul, yellow card, red card, accurate shot, percentage of the precise pass, rate of accurate access on opponent's field, the difference of ball possession percentage, shot difference, pass difference, big team superiority, home team superiority) and the number of foreign players (NoFP, active foreign players in 90 min.).

**Result:**

In the Turkish Super League, within six seasons (2014–20), the NoFP in a match increased from 10.43 (38.06%) to 17.99 (64.26%). The increment was partially linear and statistically significant (F = 594.85) in all seasons. A positive association was found between NoFP several of passes (r = 0.219), percentage of accurate passes (r = 0.133), percentage of precise passes on the opponent's field (r = 0.139), exact pass difference between opponents (r = 0.114), and ball possession difference between opponents (r = 0.113). Fouls committed decreased with the increase of NoFP (r = -0.250). Although the win probability of the teams named The Big Three (Fenerbahçe, Galatasaray, and Beşiktaş) decreased from 67% (2014 season rate) to 50% in the last two seasons, no association was identified between the NoFP and big team superiority.

**Conclusions:**

Consequently, NoFP increment may improve the game quality by increasing accurate passes and passes in the opponent's field. The number of passes and the ball possession percentage difference between opponent teams may indicate that one team has control of the game by keeping possession or the other is tactically giving possession. These findings may suggest that the game evolved into half-field, tactical, set-play, possessioning competition. Analyzing variables such as game speed, intensity, the duration of the ball in play, running distance, energy consumption, and fatigue markers may light future studies.

## Background

The percentage of foreign players in professional football teams has steadily increased over the last thirty years. This progress has been strengthened significantly after European legal and economic criteria changes [1]. The percentage of foreign players in the five major European league teams did not exceed 10% until 1985. The steady increase occurred after the year of the historical gain of the Bosman Rule. The number of foreign players (NoFP) playing in other countries rose from 18.6% to 35.6% between 1995/96/2000/01 [2].

One of the problems created by the freedom of player movement may be unfair competition due to collecting talents, especially in elite clubs participating in the Champions League. It has been revealed in some studies that the priority of drafting the players of big teams disrupts the balance between the clubs [3, 4]. When the local leagues consider, a distinct unfair competition may arise due to a few elite teams in the League transferring successful national domestic football players. Besides, it can be thought that this unfair situation arises, especially with the quota of domestic football players. A larger pool of players will improve the team's competitive balance [5].

As talented athletes tend to go to leagues with more economic superiority (Premier League, La Liga, etc.), the opportunities of young local athletes trained in these countries may reduce, and their football success in national teams may be negatively affected. However, in a study, it was found that the player mobility that occurred after the Bosman rule in 6 major European leagues did not affect the success of the national teams in the ten years. Despite this, the superiority of big groups in national leagues has climbed [6]. However, the national teams with strong leagues still reach high rankings in the World Cups [7]. In a different study, researchers stated that the imbalance between teams resulted in team revenues and the sharing of money rather than the foreign player rule [2].

**Fig. 1:**
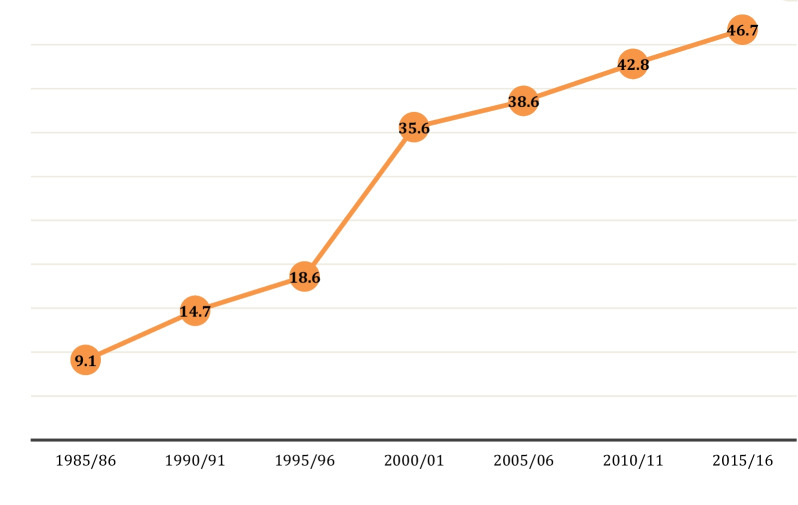
The change in the NoFP in 5 major leagues in the years between 1985 and 2016 [1]

On the negative effects of freedom of foreign players, some regulations and restraint systems that are relatively debatable have been established in the transfer system [8]. While UEFA established the "home ground rule," FIFA established the rule of 6 + 5 (the requirement that six players playing in the game must be able to play in the national team of the club's League). Several rules were established, such as the requirement that a certain number of players must be grown in that team or another club within the same national federation [2]. However, as seen in Fig. 1, there has been an increase in the NoFP in the five major leagues (England, Germany, Italy, Spain, and France), which earns the football industry [1, 9].

**Fig. 2:**
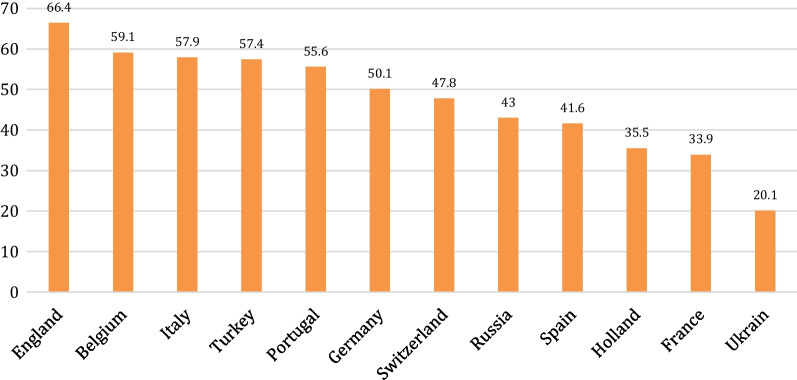
Percentage of foreign players in European leagues in 2016

The foreign player rule in the Turkish Super League has been channeled mine in the last 12 years. In the system called 6 + 0 + 4 in 2013–2014, the clubs could transfer a maximum of 10 foreigners, and a maximum of 6 pewters could enter the line-up list. Clubs would be able to transfer eight foreigners, five players would be on the field, and 3players would be on the bench in the 2014–2015 season. In 2015 – 2016, it was made a necessity to have 14 local players in 28-person squads to be determined by the football federation. This rule meant that clubs could sign up to 14 foreign players and have them all play in the starting line-up. During this period, the number of active foreign players on the teams increased by around 25%. As seen in Fig. 2, the percentage of foreign players in the Turkish Football League in 2015–2016 is higher than that of the five significant leagues shown in Fig. 1 [1].

The foreign player rule is still widely discussed in the sports media. In this respect, the number of foreign players and their effect on the game is a matter of curiosity. The Turkish League has created a suitable situation to find an answer to this question, with the number of foreign players increasing almost linearly in the last few years. In this context, this study aims to analyze the effect of foreign players on the football game longitudinally during six seasons.

## Methods

In the study, 1836 matches were evaluated within six seasons from 2014 to 2019. Data were obtained from the www.mackolik.com (OPTA Client System) website chronically each season. Inter-operator reliability of match statistics from OPTA Sports data was between 0.94 and 0.92 Kappa score, which means an excellent agreement between independent operators [10]. The variables categorized as in Table 1. "Viewing pleasure" variables were goals, shoots, crosses, passes, corners, and offsides; intense game variables were fouls, yellow cards, and red cards; "quality of the game" variables were accurate shoots, accurate passes, accurate passes in the opponent's field and percentage of precise crosses obtained (Table 1). *"C*hallenge" variables of goal difference, shoot difference, pass difference, and ball possession difference was calculated by subtraction the number of goals, shoots, passes, and ball possession percentage between the opponents. The closer these values are to zero reveal, the more significant challenge and balance between the two teams.

**Table 1:**
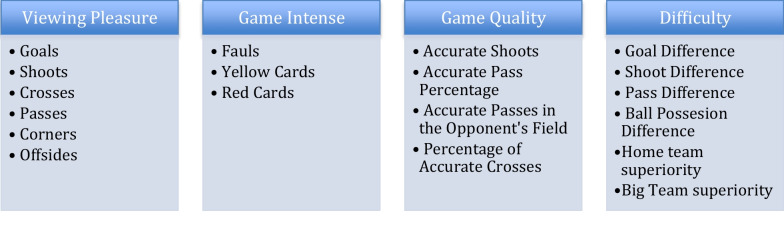
Variables whose associations with NoFP were investigated

In two variables (home team superiority, big team superiority), the win of the home team and the win of the Big Three (Fenerbahçe, Galatasaray, and Beşiktaş) teams coded as 0.Loss of points coded as 1. As the mean value of these variables approaches 1, it was understood that the point loss of these teams increased.

The study was conducted according to the guidelines of the Declaration of Helsinki and approved by the Atatürk University Review Board Approval Number: 2019–5). Atatürk University Ethics Committee approved all experimental protocols. Written informed consent was obtained from the participants to publish this paper.

### Statistical analysis

The Pearson correlation test was used to analyze the correlation between all variables in Table 1 and NoFP (total number of foreign players starting 11 + entered the game). Correlation magnitudes are interpreted using pre-specified thresholds as small < 0.20, moderate = 0.20 to 0.30, or large > 0.30, respectively [11]. A p-value less than 0.05 (typically ≤ 0.05) was statistically significant.

## Results

2014–2015 season: number of foreign players: 10,43, %37,3; 2015–2016 season: number of foreign players: 13,32, %47,6; 2016–2017 season: number of foreign players: 15,36, %54,9; 2017–2018 season: number of foreign players: 17,26, %61,6; 2018–2019 season: number of foreign players: 16,84, %60,1; 2019–2020 season: number of foreign players: 17,99, %64,3

**Fig. 3:**
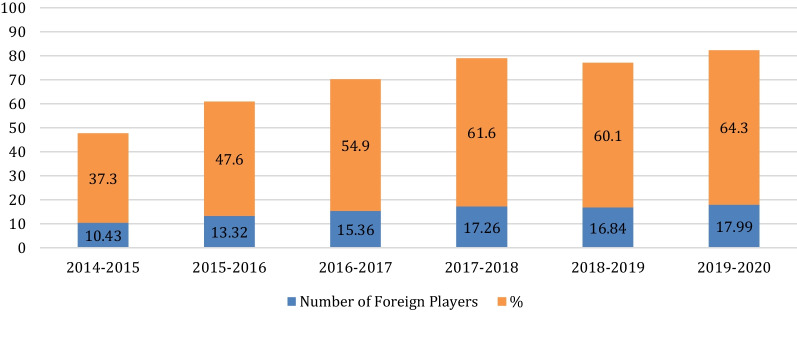
Mean and percentages of number of foreign players per game in Turkish League

The average NoFP affecting the game in a match increased from 10.43(38%) to 17.99 (64.3%) in the seasons of 2014 and 2020. An almost linear increment can be seen in Fig. 3. Although the mean NoFP values between the 2017–18 and 2018–19 seasons were not statistically different, the mean NoFP affecting the game in all the other seasons was statistically different (F = 2,436) (Fig. 3).

A statistically significant moderate positive association was found between NoFP and the passed variable (r = 0.219) (Table 2).

**Table 2:** Descriptive information and correlation table for the NoFP and viewing pleasure variables

NoFP	n	Goal	Pass	Shot	Cross	Offside	Corner
< 9	87	3.08	792	27.24	40.45	3.86	9.54
10.00	83	3.00	805	24.77	39.36	3.54	9.28
11.00	130	2.48	780	26.03	40.65	4.15	9.64
12.00	136	2.96	800	25.51	37.96	4.01	8.68
13.00	144	2.63	823	23.47	38.01	4.24	9.32
14.00	151	2.66	828	24.27	38.15	3.74	9.23
15.00	192	2.69	839	24.79	37.10	3.83	9.46
16.00	187	2.83	840	25.32	38.37	4.00	9.97
17.00	228	2.76	838	24.92	39.27	3.97	9.77
18.00	183	2.92	853	25.38	38.41	3.91	9.78
19.00	138	3.05	868	26.64	39.86	4.14	9.98
20.00	102	2.57	858	24.49	37.59	4.33	9.30
21 >	75	2.75	849	24.48	38.00	4.11	9.49
Total	1836	2.79	831	25.14	38.63	3.99	9.53
*Correlation Table*							
NoFP	r	− 0.008	0.219*	− 0.023	0.04	0.030	0.033

*The highlighted numbers indicate the significance level at 0.05

Table 3 presents the mean values and correlation coefficients of "game-intense" variables. A statistically significant moderate negative association was found between NoFP a and the total number of fouls committed (r = -0.250).

**Table 3:** Descriptive information and correlation table for the NoFP and game intense variables

NoFP	n	Fouls	Yellow cards	Red cards
< 9	87	31.57	4.74	0.17
10.00	83	32.60	4.40	0.25
11.00	130	33.15	4.75	0.36
12.00	136	32.71	4.42	0.36
13.00	144	32.28	4.42	0.31
14.00	151	32.75	4.64	0.21
15.00	192	30.15	4.50	0.28
16.00	187	30.21	4.42	0.27
17.00	228	29.66	4.53	0.34
18.00	183	28.60	4.58	0.30
19.00	138	27.50	4.37	0.29
20.00	102	28.19	4.82	0.26
21 >	75	28.20	4.64	0.25
Total	1836	30.52	4.54	0.29
*Correlation Table*				
NoFP	r	− 0,250*	0.001	0.003

*The highlighted numbers indicate the significance level at 0.05

As presented in Table 4, two small positive associations were identified between the NoFP and the "game quality" variables, which were successful passes (r = 0.133), and successful passes in the opponent's field (r = 0.139).

**Table 4:** Descriptive information and correlation table for the NoFP and game quality variables

NoFP	n	Shot on target	% Pass on target	% Pass on target on opponent's field	% Cross on target
** < 9**	87	9.67	77.02	65.90	21.54
**10.00**	83	8.75	76.45	65.02	18.41
**11.00**	130	9.07	76.03	64.91	18.42
**12.00**	136	9.35	76.08	64.82	15.49
**13.00**	144	8.31	75.63	64.06	10.13
**14.00**	151	8.56	76.31	64.85	10.30
**15.00**	192	8.32	76.792	65.25	11.91
**16.00**	187	8.99	77.09	65.77	12.31
**17.00**	228	8.97	77.50	65.94	15.52
**18.00**	183	8.85	77.50	66.60	16.04
**19.00**	138	9.57	78.67	67.74	18.79
**20.00**	102	8.43	77.73	66.46	17.60
**21 > **	75	8.76	78.45	67.45	19.22
**Total**	1836	8.87	77.01	65.71	15.15
*Correlation Table*					
**NoFP**	**r**	− 0.020	0.133*	0.139*	0.005

*The highlighted numbers indicate the significance level at 0.05

Table 5 reveals the correlation of "challenge" variables, including the number of goals, shots, passes, and ball possession difference between the opponents and the home and Big Three Team superiority. There were small positive associations between pass difference (r = 0.114) and ball possession difference variables with NoFP identified (r = 0.114 and 0.079, respectively). No associations were found between NoFP—Home team superiority and NoFP—Big Three Team superiority. Interestingly, despite this finding, the probability of losing points for the big teams has increased from 33% the o 50% in the last two seasons.

**Table 5:** Descriptive information and correlation table for the NoFP and challenge variables

NoFP	n	Goal difference	Shot difference	Pass difference %	Ball possession percentage difference %	Big three team superiority (n = 536)	Home team advantage
** < 9**	87	1.55	5.05	123.73	15.44		
**10.00**	83	1.36	7.18	109.50	13.67		
**11.00**	130	1.12	6.75	129.21	16.39		
**12.00**	136	1.73	7.92	159.51	18.37		
**13.00**	144	1.59	7.41	174.12	20.05		
**14.00**	151	1.50	6.03	175.57	19.21		
**15.00**	192	1.48	7.79	178.01	21.05		
**16.00**	187	1.69	7.14	191.77	21.69		
**17.00**	228	1.27	6.70	171.88	19.97		
**18.00**	183	1.53	6.59	174.20	19.95		
**19.00**	138	1.42	7.21	205.59	24.18		
**20.00**	102	1.33	8.24	191.18	21.52		
**21 > **	75	1.56	6.40	189.32	21.42		
**Total**	1836	8.87	77.01	65.71	15.15		
*Correlation Table*							
NoFP	R	− 0.013	− 0.009	0.114*	0.079*	-0.045	0.002

*The highlighted numbers indicate the significance level at 0.05

## Discussion

When the literature is reviewed, it can be observed that the findings of studies differ [3, 5, 12]. The balance between teams and foreign player mobility has been analyzed in this study. To determine the compensation, analyses have been made within the framework of some models. Different models have revealed different results [5]. In one study, the balance among teams has been adjusted according to the score distribution model and team rank of the seasons 1986–96 (Pre-Bosman) and 1997–2005. According to findings, the dominance of top European clubs has fallen in the years following Bosman. In 17 leading European leagues, it was presented that player mobility has been shown to increase competitive balance due to the increase in the NoFP joining the pool to which talented players can be transferred [5]. In another study, the protective rules and suggestions of UEFA and FIFA have been discussed. While it has been argued that the protective laws put by these two football leaders have conflicted with each other, it has been stated that the increase in the NoFP as a result of the effect of globalization has contributed to the talent distribution and the development of the football industry globally [2]. According to a not football-related study, it was stated that a historical effect was observed in the Major Baseball League after racial discrimination disappeared and foreign players started to play [13]. In another study, the balance among teams was calculated through the effect of the team's position in one season to the other; no difference was found between before and after Bosman [12].

Furthermore, in a study evaluating 7 European leagues, it has been revealed that after the Bosman rule and the establishment of the Champions League, the competitive balance in national leagues has not changed. Still, the difference in international quality has increased [3]. In some studies, the positive performance effects of the NoFP on the team in local and international football competitions are mentioned [14–16]. In one study, in which the team age and different variables were examined, it was observed that talented foreign players contributed significantly to their teams in the German Football League [17]. In a study about foreign football players in the English football league, while the importance of raising young football players has been discussed, it has been stated that foreign football players with potential have been positively affected by the football culture of England [18]. With the introduction of the Bosman rule, foreign players of different races have contributed to their teams on other variables and developed the commercial volume of football worldwide. Looking over the five main leagues, it has been seen that the football players who scored the most goals in the English League have been foreign football players [19]. In a large-scale study conducted on over 1000 football clubs worldwide, it has been stated that foreign football players have contributed positively to their clubs [16]. Considering the European continent, foreign football players playing in different leagues have not negatively affected the success of national football teams in terms of international football player trade [20]. A similar positive effect seems to be valid in other continent football leagues. For example, in the Chinese football league, one study revealed that foreign players have been better than local players in terms of passing, shooting, positioning, and general physical capacity [21].

As discussed below, our study highlights foreign players' inventive contribution. NoFP in the Turkish Super League has shown an increase of 27% in the last six seasons. This increment can be interpreted as significantly affecting the game style, especially by affecting the pass and the variables related to the pass and ball possession. Fortunately, this rise in the number of passes appears as an increase in accurate passes and accurate passes on the opponent's field. This finding confirms that the number of lost balls (turnovers) decreased, and the passing quality, regardless of quantity, improved. "Possession t the contest" is a new statistical metric introduced in Qatar World Cup 2,022, designed to measure the proportion of the game when neither side is in control of the ball. Including this variable in future studies may clarify the impact of NoFP on the processioning issue. That can be hypothesized that this variable would decrease with the increment of in NoFP.

A study comparing the physical and technical characteristics of local and foreign players in the Chinese League has observed that foreign players have been more successful in positioning and passing performance [21]. A study of 346 Chinese football players indicates that foreign players have been more successful than local players, especially midfielders and strikers, in terms of passing performance [22]. In a study where technical analysis of the Champions League performances of teams from five major European football leagues (Ligue 1, France; Bundesliga, Germany; Premier League, England; La Liga, Spain; and Seria A, Italy) has been performed, it has been determined that meeting with the ball and passing percentages of Serie A players have been lower than the other leagues [23]. However, it has been stated that among twenty different variables acquired from the five major European football leagues, no significant relationships exist between these variables and passes [23]. In another similar study on the Chinese football league [24], it was observed that foreign players have been more successful, especially in passing performance over 49 determinant performance variables. In light of the research mentioned above findings, when the results of our study have been compared to the literature [21, 24], the Turkish Super League and the Chinese football league have shown similarities in terms of the effects of foreign players in domestic leagues through their pass performances. Except for Chinese studies, only one study analyzed the impact of a player's nationality on a match [25]. Specifically, Bush et al. reported that technical data revealed trivial or minor differences between U.K. and non-UK players in the EPL. Consistent with our findings, the non-UK players performed more passes in 2006–2007 than the U.K. players. But according to the longitudinal data, match performance characteristics in the English Premier League (EPL) were similar between the local and foreign populations [25]. Besides, The EPL has undergone substantial change over the last decade, with the distances covered at high intensity and sprinting increasing by 30–50% and the number of passes rising by 40%. However, a lack of supporting evidence, a commonly held belief within the game, was that the increased migration of ton-UK players into the EPL could account for these recent performance alterations [26, 27].

Likewise, the *number of pass differences* and *ball possession percentage differences* between opponents increased with NoFP in our study. This finding may suggest that the game has been less competitive. From the authors' point of view, it should be considered an adjustment of the game tactically. The tactical tendency of one team to have possession or one to give possession to the other appeared to be increased. In this respect, it can be thought that the increase in the NoFP has affected the mentality of the game. It is well known that the mentality of play (keeping possession, long pass play, short play, counter-attack play, etc.) has changed in different leagues [23, 27]. The association of passing and ball possession with NoFP, which emerged in our study, may have improved the game. One study analyzed 380 matches in the Spanish football league (La Liga) in the 2018–2019 season through performance parameters. According to this research, a significant positive correlation has been found between the number of passes and goals scored. Especially in the first half of the matches, as the number of passes has increased, the number of goals also has increased [28].

Another study found a moderate negative association between NoFP and fouls committed (r = -0.250). In this regard, it can be said that the ball-in-playtime has increased. It can be seen that the average number of fouls per match in the Turkish Super League (mean value of 6 seasons is 30.52) has been well above that of the major European leagues, but this difference has substantially decreased with the rise in the NoFP. According to the CIES Football Observatory [1], the average number of fouls per season has ranged from 20.9 in the Premier League to 27.0 in the La Liga. This figure has been 24.0 in Bundesliga, 26.4 in Ligue 1, and 26.6 in Serie A. According to the authors, this significant difference may reflect, in part, the existence of different referee styles. In addition, the increment of the difference between the opponents in *ball possession percentage*, *number of passes,* and *accurate passes in the opponent's half* has shown that the game was played in a smaller area. The closer game format set plays and narrowed field may have caused the decrease in the number of fouls (decreased sprint distance and empty spaces). In addition, it was observed that the reduction in the number of fouls was not reflected in the number of cards. This indicates that decreased number of fouls were no-card fouls. It can be thought that the number of easy fouls by the referees has reduced.

Although the probability of losing points for big teams has increased in six seasons, this change has not been statistically significant in the relationship between the NoFP and the superiority of The Big Three (Table 5, r = -0.045). Likewise, the home team's dominance has not been affected by the increase in the NoFP. One study has found that the home team has committed fewer fouls and received fewer yellow and red cards than the away team and that an inverse trend emerged for the game's intense variables. The inner field positively affects the player level among variables of goal number, total shots, shots on goal, and assists. Besides, while the home-field advantage effect has been observed for goals scored by foreign players, local players have scored an equal number of goals at home and away [29].

## Conclusion

In this study, it can be said that the number of passes in the opponent field number of passes on target has increased the quality of games with the increase in the NoFP in the Turkish Super League. Also, the increase in the passes between the two teams and the difference in ball possession indicate that teams have changed their game mentality (the tendency to keep possession–the tendency to give the ball to the opponent) offensively and defensively. According to these findings we can say that the increment in NoFP has turned football into pass-based, pocssessioning, set play game.. It may be thought that the coaching skills, players' game insight, tactical setups, and field formations/positioning have become an essential part of Turkish football.

The variables used in this study may have been partially insufficient to explain the effect of the change in the NoFP. For a more valid analysis, values such as game intensity, running distance, number of sprints, oxygen consumption, and heart rate may need to be obtained. Likewise, modeling in which standard performance parameters are used for comparative analysis of different countries can contribute to the literature. It is thought that researching the effects of foreign players in other countries with various performance parameters will contribute to the literature.

## Data Availability

The data presented in this study are available on website: https://osf.io/gdn5k/ with Identifier: https://doi.org/10.17605/OSF.IO/GDN5K.
